# Direct estimation of cause-specific mortality fractions from verbal autopsies: multisite validation study using clinical diagnostic gold standards

**DOI:** 10.1186/1478-7954-9-35

**Published:** 2011-08-04

**Authors:** Abraham D Flaxman, Alireza Vahdatpour, Spencer L James, Jeanette K Birnbaum, Christopher JL Murray

**Affiliations:** 1 Institute for Health Metrics and Evaluation, University of Washington, 2301 Fifth Ave., Suite 600, Seattle, WA 98121, USA; 2 University of Washington, Department of Health Services, Seattle, USA

**Keywords:** Verbal autopsy, cause of death certification, validation, direct estimation

## Abstract

**Background:**

Verbal autopsy (VA) is used to estimate the causes of death in areas with incomplete vital registration systems. The King and Lu method (KL) for direct estimation of cause-specific mortality fractions (CSMFs) from VA studies is an analysis technique that estimates CSMFs in a population without predicting individual-level cause of death as an intermediate step. In previous studies, KL has shown promise as an alternative to physician-certified verbal autopsy (PCVA). However, it has previously been impossible to validate KL with a large dataset of VAs for which the underlying cause of death is known to meet rigorous clinical diagnostic criteria.

**Methods:**

We applied the KL method to adult, child, and neonatal VA datasets from the Population Health Metrics Research Consortium gold standard verbal autopsy validation study, a multisite sample of 12,542 VAs where gold standard cause of death was established using strict clinical diagnostic criteria. To emulate real-world populations with varying CSMFs, we evaluated the KL estimations for 500 different test datasets of varying cause distribution. We assessed the quality of these estimates in terms of CSMF accuracy as well as linear regression and compared this with the results of PCVA.

**Results:**

KL performance is similar to PCVA in terms of CSMF accuracy, attaining values of 0.669, 0.698, and 0.795 for adult, child, and neonatal age groups, respectively, when health care experience (HCE) items were included. We found that the length of the cause list has a dramatic effect on KL estimation quality, with CSMF accuracy decreasing substantially as the length of the cause list increases. We found that KL is not reliant on HCE the way PCVA is, and without HCE, KL outperforms PCVA for all age groups.

**Conclusions:**

Like all computer methods for VA analysis, KL is faster and cheaper than PCVA. Since it is a direct estimation technique, though, it does not produce individual-level predictions. KL estimates are of similar quality to PCVA and slightly better in most cases. Compared to other recently developed methods, however, KL would only be the preferred technique when the cause list is short and individual-level predictions are not needed.

## Background

In settings where a non-negligible proportion of the population dies outside of the hospital system, verbal autopsies (VAs) are emerging as a vital tool for understanding the population-level patterns of cause-specific mortality fractions (CSMFs). By combining this with robust information on levels of age-specific all-cause mortality (also collected through household surveys, e.g., of sibling survivorship), it is possible to estimate age- and cause-specific mortality rates. Most population-level estimates derived from VAs are created in two phases, by first assigning a cause or several causes to each death and then calculating CSMFs from the number of deaths or partial deaths assigned to each cause. Direct estimation is an alternative approach that produces population-level estimates of CSMFs directly from the VAs without the intermediate stage that requires assigning deaths to each VA. The direct estimation method proposed by King and Lu (which we will call the KL method) is designed to capture complex patterns of interdependence between various signs and symptoms in the VA instrument [1,2]. This approach can be interpreted as a sophisticated multiclass generalization of the classic back-calculation approach of epidemiology and has been shown to be a promising method in theoretical simulation and small-scale validation studies [2].

The KL method is based on the following matrix expression:

Where *P*(**S**) is the distribution of symptom profiles in the test dataset, *P*(**S**|*D*) is the distribution of symptom profiles for each cause of death (calculated using the training dataset), and *P*(*D*) is the distribution of causes of death in the test dataset. A symptom profile is a combination of *k *different symptoms. Each symptom is dichotomous, so *k *symptoms yield 2^k ^symptom profiles. *P*(**S**) and *P*(**S**|D) are calculated by tabulation. For a symptom profile *s*_0_, *P*(**S **= *s*_0_) is calculated by counting the fraction of VAs to be analyzed that endorse symptom profile *s*_0_. For a symptom profile *s*_0 _and cause *j, P*(**S **= *s*_0_|D = j) is calculated by counting the fraction of VAs in the "training set" with disease j as the cause of death that endorses symptom profile *s*_0_. Quadratic programming or least squares approaches may be used to solve this equation. King and Lu reported that the expected value of CSMFs estimated by their direct estimation method in repeated samples yields plausible CSMFs in a simulation study using data for 13 adult causes of death in China and 11 causes of child death in Tanzania. King and Lu [1] further stress that the direct CSMF estimation approach does not depend on the presence in the VA instruments of items with high sensitivity or specificity for particular causes. They argue the approach provides an efficient, low-cost approach for estimating CSMFs and they derive analytical strategies for choosing symptoms from an instrument that will optimize performance. At least two studies have taken the KL method and applied it to real-world verbal autopsy datasets [3,4].

Despite the impressive results with small errors in CSMFs reported by King and Lu, there are several outstanding issues that need to be understood before widespread adoption of the method. First, King and Lu report in repeated experiments the expected value of the CSMF produced by their method compared to the true CSMFs using test and train datasets. They do not report a metric of the average error in CSMFs across repeated experiments, leaving it unclear how well the method will work in a given real-world application. Second, in all of the cases that they report, the CSMF composition of the train and test datasets are either identical or very close to each other. The performance of the KL method when the CSMF composition of the training set is different than the test dataset has not been established. Third, the validation data reported by King and Lu pertain to relatively short cause lists of length 11 and 13, respectively. The performance of the KL method for the longer cause lists desired in most VA studies has not yet been established. Fourth, until recently [5] there have been no standardized metrics to compare the performance of different VA methods for the estimation of CSMFs, limiting the comparison of KL to other methods such as PCVA, InterVA, Symptom Pattern, or others [6-8].

In this paper we present the results of a validation study of the KL method, using a large dataset with a realistically diverse cause list collected in the Population Health Metrics Research Consortium (PHMRC) gold standard verbal autopsy validation study [9]. The study was undertaken to develop a range of new analytical methods for verbal autopsy and to test these methods using data collected in six sites in four countries (Mexico, Tanzania, India, and the Philippines). The study is unique, both in terms of the size of the validation dataset (7,836, 2,075, and 2,631 deaths in adults, children, and neonates respectively) and the use of rigorously defined clinical diagnostic criteria for a death to be included in the study as a gold standard cause of death. The dataset collected through the PHMRC is sufficiently large to be able to explore the relationship between CSMF errors by cause and overall CSMF accuracy and the size of training and test datasets.

## Methods

**Figure 1:**
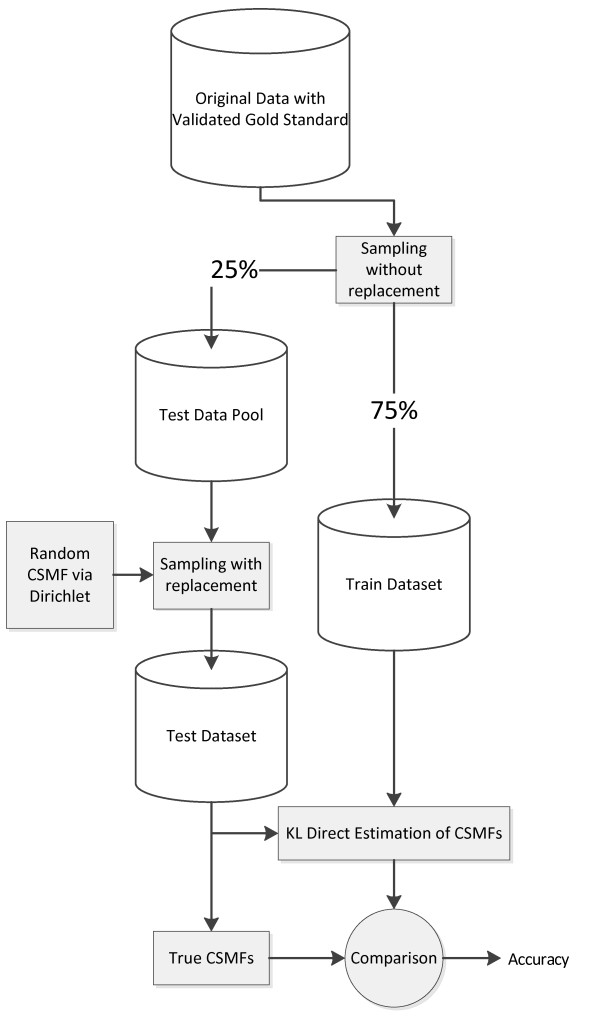
**The process of generating 500 test and train datasets and applying KL estimation to them**. After dividing the whole dataset into 25% testing and 75% training portions (randomly, stratified by cause), a draw from an uninformative Dirichlet distribution was used to perturb the cause combination of the test set (by resampling each cause with replacement according to a CSMF that was drawn from Dirichlet distribution). Accuracy of the KL method was calculated by comparing the KL-estimated CSMFs and the true CSMF of the test dataset.

We use the PHMRC gold standard VA dataset to undertake three distinct analyses to understand the performance of the KL method in different settings. Details of the methods used for establishing the gold standard cause of death and for the collection of the VA data are reported elsewhere in detail [9]. The PHMRC instrument uses separate modules for neonate, child, and adult deaths so these sets of deaths have been analyzed separately. The final cause lists are mutually exclusive and collectively exhaustive for all causes, and contain 11 causes for neonates, 21 causes of child death, and 34 causes of adult death. The development of training and test datasets is described in detail elsewhere [9] and is summarized in Figure 1.

Figure 1 outlines the basic simulation design to generate a range of test and training datasets. First, for each cause we split the data randomly without replacement, with 75% into a training set and 25% into a test set. This step was repeated 500 times to avoid results being influenced by the idiosyncrasies of a particular data split. We then sampled CSMF compositions from an uninformative Dirichlet distribution and randomly resampled (with replacement) the available deaths in the test set to generate a test dataset with the prescribed total number of deaths and CSMF composition. By varying the CSMF compositions of test datasets as well as the total number of deaths, we generated a wide array of validation datasets. Each one maintained a strict separation of training and test data, which guarantees that our metrics are for "out-of-sample" prediction quality. This method generates test/train datasets with independent CSMF composition.

Over the course of the PHMRC gold standard VA validation study, it became clear that metrics for gauging the quality of VA methods are quite subtle and are not standardized between research efforts. The complex issues are described fully by Murray et al. [5], who also proposed new metrics that allow for quality comparison across cause lists and cause compositions. Following their recommendations, we report median CSMF accuracy across 500 test datasets. At the cause-specific level we report the intercept, slope, and root mean squared error (RMSE) for the relationship between estimated CSMF and the true CSMF assessed using linear regression.

Murray et al. [10] showed that in China, the recall of the household or possession of medical records recorded in the VA interview had a profound effect on both the concordance for PCVA as well as the performance of the computer-coded VAs. However, obtaining useful information from this health care experience (HCE) cannot be assumed for many settings where VA will be used. Therefore, we identified all signs and symptoms that we suspected could be much more informative for people who have received health care and performed all validation experiments on two versions of the datasets developed above, one with all variables (noted as *with HCE*) and one version excluding recall of health care experience (*without HCE*).

### Validating KL CSMFs for neonates, children, and adults

In the first test, we apply the KL software to the 500 pairs of training and test datasets for each of the three age groups. We assess the performance of the KL method by reporting median CSMF accuracy and the relationship between the estimated CSMFs and true CSMFs by cause. The KL method requires the user to select two parameters: the number of symptoms to be subset from all symptoms (*nSymp*), and the total number of draws of different subsets (*n.subset*). For these main results, we used settings of 10 symptoms and 400 iterations.

We also investigated the effect of these parameters on the accuracy of the KL method by an extensive exploration of the range of settings. We repeated our assessment while varying the *nSymp *from eight to 18. We also varied *n.subset *from 200 to 600.

### Assessing the relationship between KL CSMF accuracy and the number of causes

To evaluate the dependence of the method's CSMF accuracy on the number of causes in the cause list, we performed the following experiment. For *n *= 5, 6, ..., 46 we randomly chose *n *causes of death and used a CSMF drawn from an uninformative Dirichlet to construct a test dataset that contains exactly *n *causes of death. (The maximum is 46, as our original adult dataset has 46 causes of death.) The deaths were sampled from the original 25% test and 75% train pool datasets described above. We performed 500 iterations for each *n*. By the nature of this test, the number of deaths in the train and test datasets do not vary as the number of causes are altered. This provides a direct assessment of performance strictly as a function of the number of causes.

### Assessing if KL accuracy is influenced by the correlation between training and test dataset CSMF composition

The technique described for the experiments above generates test and training sets that have independently random CSMFs. We suspected that the KL performance in previous studies has been exaggerated because the CSMF compositions of test and train datasets have been similar. To investigate this hypothesis, we conducted an additional analysis using training and test sets generated by sampling deaths from training and test pools uniformly at random (with replacement). In contrast to previous experiments in which the CSMFs of the test and train datasets are independent, the test and train datasets in this case both have CSMF combinations similar to those of the original pool. The same metrics are used for this assessment.

## Results

**Figure 2:**
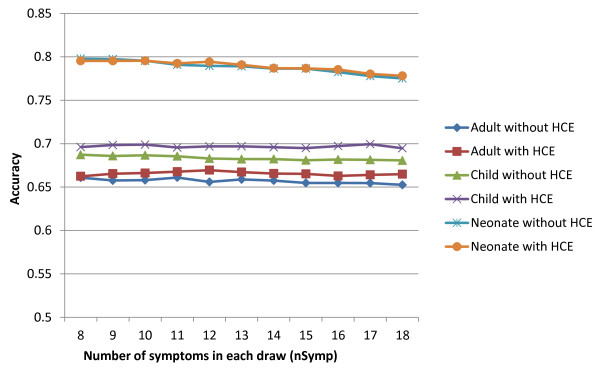
**Variation of CSMF accuracy of the KL method as a function of symptom cluster size (*nSymp*)**. For all age groups, with and without HCE, varying the symptom cluster size had little effect on CSMF accuracy.

CSMF accuracy of KL for adult, child, and neonatal VA analysis was found to be largely independent of using different sized symptom clusters and including or excluding HCE (Table 1 and Figure 2). For all experiments, *n.subset *of KL method, which specifies the total number of draws of different subsets of symptoms, is set to 400. Through our experiments we saw no significant variation in the CSMF estimation accuracy by changing the symptom cluster size when *n.subset *is large enough (greater than 200). Figure 2 shows the variation of CSMF accuracy when the symptom cluster size is varied between eight and 18. (The KL method requires that the number of causes in the module be fewer than the number of symptom profiles 2^k^. Hence, theoretically k = 6 is the smallest allowed. In addition, since some symptom profiles never appear in the data, k = 8 is the smallest *nSymp *we could use for all adult, child, and neonate datasets.)

**Table 1:** Median CSMF Accuracy for KL and PCVA, by age group with and without HCE

		KL		PCVA	
		Median	95% UI	Median	95% UI
**Adult**	**No HCE**	0.661	(0.654, 0.665)	0.624	(0.619, 0.631)
	**HCE**	0.669	(0.664, 0.673)	0.675	(0.669, 0.680)
**Child**	**No HCE**	0.687	(0.682, 0.692)	0.632	(0.626, 0.642)
	**HCE**	0.698	(0.692, 0.702)	0.682	(0.671, 0.690)
**Neonate**	**No HCE**	0.797	(0.784, 0.805)	0.695	(0.682, 0.705)
	**HCE**	0.795	(0.783, 0.806)	0.733	(0.719, 0.743)

As shown in Table 1, without HCE the KL method slightly outperforms PCVA. We remark that the PCVA accuracy for child VAs in absence of HCE variables is 0.05 below the median KL accuracy. For neonatal VAs without and with HCE variables, the KL method CSMF accuracy is 0.797 (95% uncertainty interval [UI]: 0.784, 0.805) and 0.795 (0.783, 0.806), respectively, which are also substantially higher than than CSMF accuracy of PCVA.

The relationship between estimated and true CSMFs for each cause in adults, children, and neonates are shown in Additional file 1. A good estimation should have intercept close to zero and slope close to one. With slope 0.631, intercept 0.015, and RMSE 0.013, drowning is the most accurately estimated cause of death in adult VA. In the same module, stomach cancer and other cardiovascular diseases are the least accurately estimated causes with slope being approximately 0.08. Other cardiovascular disease also has a high intercept (0.047), which shows it is substantially overestimated when the true CSMF is low. In the child module, violent death is the most accurately estimated CSMF with slope 0.480, intercept 0.024, and RMSE 0.016, and other digestive disease is the worst estimated cause where slope, intercept, and RMSE are 0.092, 0.031, and 0.010, respectively. In the neonatal module, stillbirth is almost perfectly estimated with slope, intercept, and RMSE being 0.98, 0.003, and 0.017, respectively. Pneumonia has the lowest accuracy of estimation with a slope, intercept, and RMSE of 0.199, 0.053, and 0.026. As it is observed, the quality of prediction is generally higher in neonatal module. It is observed that for causes for which estimation is not accurate, KL tends to assign close to constant cause fractions, which results in higher intercepts and lower slopes. As a result, small CSMFs are overestimated and large CSMFs are underestimated in such causes.

We found that in adult VA, the KL method is most effective in predicting CSMF for maternal causes and causes that are due to injuries, such as drowning. In child VA, measles, malaria, bite of venomous animal, and violent death were most accurately predicted. For neonatal VA, stillbirth and preterm delivery cause group were best. In contrast, KL performs poorly in predicting stomach cancer and other noncommunicable disease in adults, other digestive disease and other infectious disease in children, and pneumonia in neonates.

As shown in Table 1, in general, the effect of the HCE variable on the accuracy of CSMF estimation is not large (the change is 0.008, 0.011, and -0.002 for adult, child, and neonates). For the majority of causes in all age groups, accuracy slightly increased when HCE variables were added; however, the change was not large. For example, in the adult module, average slope increases from 0.236 to 0.247 and average intercept decreases from 0.024 to 0.023 (mean RMSE does not change).

**Figure 3:**
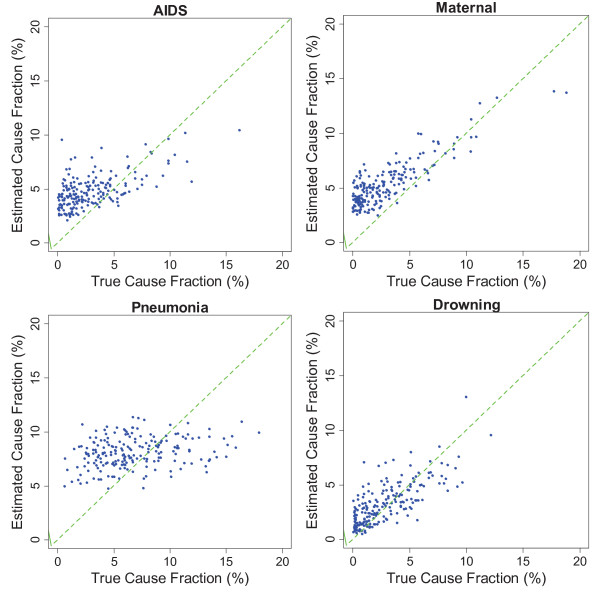
**Estimated versus true cause fractions for AIDS, maternal, pneumonia, and drowning in adults in 500 random resamplings of the validation dataset**. Causes like pneumonia were overestimated when rare but underestimated when common, while causes like drowning were estimated with accuracy that does not depend closely on true cause fraction.

**Figure 4:**
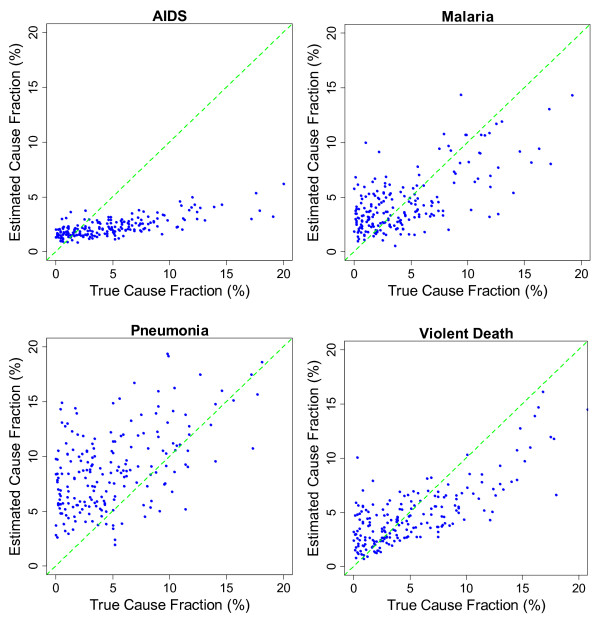
**Estimated versus true cause fraction for AIDS, malaria, pneumonia, and violent death in children in 500 random resamplings of the validation dataset**. These causes were underestimated when rare and overestimated when common.

**Figure 5:**
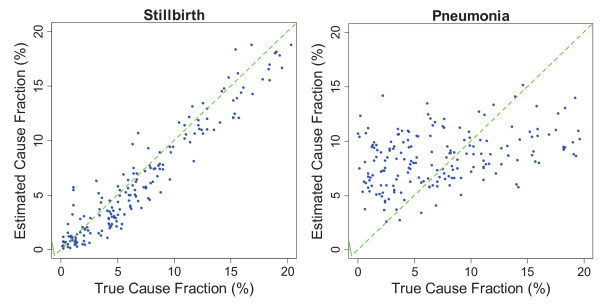
**Estimated versus true cause fraction for stillbirth and pneumonia in neonates in 500 random resamplings of the validation dataset**. Stillbirth estimations were highly accurate, while pneumonia was either underestimated or overestimated in most cases.

Figures 3, 4, and 5 show the estimated and true CSMF of a selection of causes in the three age groups. A lower slope in the regression shown in Additional file 1 shows more deviation from the perfect estimation line in the figures. We found that KL tends to equally distribute deaths among causes, which overestimates the CSMF when the true CSMF is very low and underestimates when it is high.

**Figure 6:**
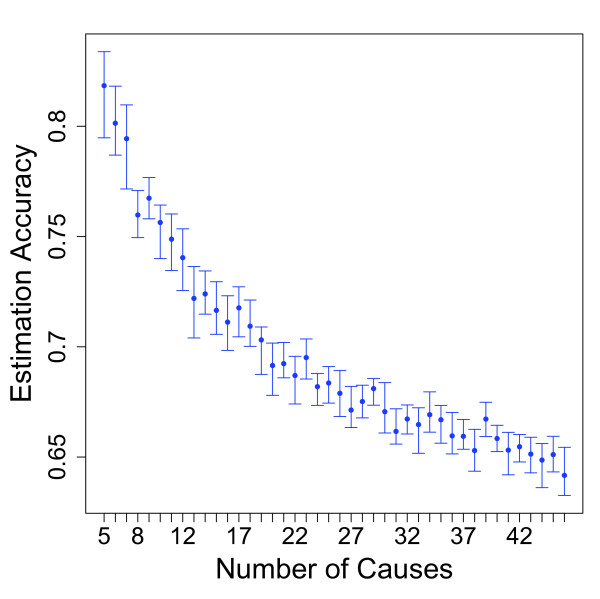
**Median CSMF accuracy versus number of causes on a cause list for the KL method**. The test datasets for this experiment were generated by randomly selecting a set of causes and constructing test datasets using an uninformative Dirichlet distribution. The KL method has excellent performance for short cause lists, but rapidly degrades as the length of the list increases.

As shown in Figure 6, the number of causes on the cause list has a very large impact on the accuracy of KL CSMF estimations. While these results are acquired by randomly dropping causes from the adult module, a comparison with the neonate and child modules' accuracy results (Table 1) suggests that the most important parameter in the KL method's superior performance in child and neonate modules is the lower number of causes in these modules. Accuracy is above 0.75 when the cause list contains fewer than 12 causes. For larger cause lists, such as those used for practical applications in adults and children, the KL method generates progressively lower levels of CSMF accuracy.

We found that KL is extremely sensitive to the level of similarity between cause composition in the train and test datasets. We observed that if both test and train sets are randomly sampled with the same cause composition, KL estimation will yield dramatically higher CSMF accuracy. For example, for adult VAs with HCE when the test and train set have the same CSMF, the median CSMF accuracy is 0.947 (0.945, 0.951), which is 0.28 points higher than the accuracy of KL for redistributed test sets and within 0.05 of the maximum possible accuracy.

## Discussion

In this first large-scale validation of the KL method for direct CSMF estimation compared to gold standard cause of death assignment, we found that the method performs about as well as PCVA in terms of CSMF accuracy. Compared with some new methods [8,11,12], KL generates substantially less accurate CSMFs for adults and children. The KL method yields CSMF estimates that tend to be biased upwards when the true CSMFs in the test datasets are low and biased downwards when the true CSMFs are high. The extent of these biases is highly variable across causes. The biases in the KL estimates of CSMFs bear considerable resemblance to the biases observed in PCVA by cause, although there is some variation in performance by cause.

Our findings contradict several previous claims about details of the method. First, we found that varying symptom cluster size from eight to 18 made essentially no difference to the results. Second, KL does well in estimating CSMFs for causes such as road traffic accidents and drowning for which there are sensitive and specific symptoms. These are the same causes on which physicians also perform well. Our experiments show that, similarly to individual-level cause assignment techniques, KL is inaccurate in finding CSMFs for causes with weak symptom presence. Where there is not a clear set of sensitive and specific symptoms, the KL method tends to yield CSMF estimates that are biased towards the cause fraction in the training dataset rather than the test dataset. This tendency of the KL method to project the training dataset CSMF onto the test dataset is confirmed by the experiment in which we found that KL accuracy was exaggerated when the training and test datasets have identical CSMF compositions.

One clear advantage of KL compared to PCVA is in the tests in which household recall of health care experience is excluded from physician review and the KL method. Thus, in settings where populations are expected to have little exposure to health care, the KL approach should be preferred to PCVA. This finding, however, must be tempered with the comparison to other methods (Symptom Pattern, Tariff, and Machine Learning) that all have better performance than KL in the absence of household recall of health care experience.

The relatively disappointing performance of KL compared to published claims will surprise some readers. The key explanation is the number of causes included in our study for adults and children. Our finding that the KL method's accuracy dramatically decreases as the number of causes increases explains why KL has performed well in previous validation studies (e.g., [2]). These have all used lists of causes that contain fewer than 15 causes. For studies with smaller number of causes (e.g., neonatal VA studies usually consider fewer than eight to 10 causes of deaths) our findings suggest that the KL method produces very good results with a CSMF accuracy greater than 0.75. A further reason for the exaggerated performance previously reported for KL may be that previous studies used test and train datasets that had similar CSMF compositions. Our experiments here show that the KL method in this special case yields substantially higher levels of CSMF accuracy. In real populations, there is no reason to expect that a training dataset collected in a hospital will have the same CSMF composition as the population. In fact, a method that largely returns the training dataset CSMF composition adds little information beyond the CSMF composition of the training dataset. Thus, a more realistic assessment of KL performance follows from the cases in which the CSMF compositions in the test and train datasets are unrelated.

A central assumption of the KL approach is that, conditional on the cause of death, the symptom profiles of reference deaths, usually from hospitals, are the same as community deaths. The data in the PHMRC study was collected from deaths that met stringent gold standard diagnostic criteria, and most of these necessarily occur within the hospital system (community deaths simply cannot meet the diagnostic criteria for many causes). As a result, this validation study cannot directly investigate the importance of this assumption to the KL method. However, by excluding HCE variables from the study, we have emulated this setting and found little change to our results.

## Conclusion

Our validation of the KL method for direct estimation of CSMF from VA data collected in the PHMRC study showed that KL performs at about the same level as PCVA for adults, slightly better for children, and much better for neonates. Since it is a direct method, it does not yield cause of death assignments for individual deaths. We also found that KL performance is sensitive to the number of causes on the cause list, and as the number of causes under consideration increases, the quality of KL estimation decreases precipitously. This degradation is especially relevant when using VA to understand population-level patterns of adult mortality, in which the accuracy of KL becomes comparable to PCVA. Thus we judge KL to be a reasonable approach for neonatal VA and other settings with very short cause lists, but not as useful in its current form for adult or child VA. For adults and children, other methods, such as the Simplified Symptom Pattern, Random Forest, and Tariff, have better CSMF accuracy and also provide individual death cause assignment.

## Abbreviations

CSMF: cause-specific mortality fraction; KL: King and Lu cause-specific mortality fraction direct estimation method; PCVA: physician-certified verbal autopsy; PHMRC: Population Health Metrics Research Consortium; RMSE: root mean squared error; HCE: health care experience; VA: verbal autopsy

## Competing interests

The authors declare that they have no competing interests.

## Authors' contributions

AV performed analyses and helped write the manuscript. SLJ and JKB helped in data preparation and preliminary studies. CJLM designed the study and drafted the manuscript. ADF contributed in the study design, edited the manuscript, and approved the final version. ADF accepts full responsibility for the work and the conduct of the study, had access to the data, and controlled the decision to publish. All authors have read and approved the final manuscript.

## Supplementary Material

Additional file 1 **Slope, intercept, and RMSE from linear regression of estimated versus true CSMFs, by age group and cause with and without HCE**. Click here for file
